# Mental Health Screening of Healthcare Professionals Who Are Candidates for Psychological Assistance during the COVID-19 Pandemic

**DOI:** 10.3390/ijerph182111167

**Published:** 2021-10-24

**Authors:** Bernat-Carles Serdà, Maria Aymerich, Josefina Patiño-Masó, Mònica Cunill

**Affiliations:** 1 Health and Healthcare Research Group, Health Sciences Department, Girona Biomedical Research Institute (IDIBGI), University of Girona, 17071 Girona, Spain; 2 Faculty of Education and Psychology, Quality of Life Research Institute, University of Girona, 17071 Girona, Spain; maria.aymerich@udg.edu (M.A.); monica.cunill@udg.edu (M.C.); 3 Nursing Department, Quality of Life Research Institute, University of Girona, 17071 Girona, Spain; josefina.patino@udg.edu

**Keywords:** healthcare professionals, COVID-19, mental health, anxiety, depression, emotional regulation

## Abstract

Healthcare professionals (HCPs) are among those most affected by the COVID-19 health emergency, with many presenting symptoms of anxiety and depression. Research shows that one of the factors involved in mitigating the impact of stressful situations is the use of cognitive emotional regulation mechanisms. The aims of this study were (a) to describe the functional and dysfunctional cognitive emotional regulation mechanisms (FRMs and DRMs) by gender, (b) to screen the main group of healthcare professionals who are candidates to receive psychological assistance based on FRMs and DRMs, and (c) to determine the HCP profile of candidates for psychological assistance. A cross-sectional descriptive study was conducted. Data were obtained from an adhoc questionnaire—the Cognitive Emotional Regulation Questionnaire (CERQ-18), the Generalized Anxiety Disorder scale (GAD-7), and the nine-item Patient Health Questionnaire (PHQ-9). The representative sample comprised 1452 HCPs. The results revealed significant differences between men and women in the use of DRMs. Women showed a higher use of catastrophizing (≤0.001) and rumination (0.008). The screening procedure detected that 7.5% (109 cases) of the HCPs were candidates to receive psychological support. According to the results of this study, age group (30–39 years old), professional activity (being a nurse or nursing assistant), and having psychological symptoms of anxiety and depression are variables that independently increase the probability of requiring psychological assistance. The gender variable was not found to be an independent factor when it comes to receiving psychological support. In conclusion, it is necessary to consider the influence of cognitive emotional regulation strategies employed by HCPs in the screening of candidates for psychological assistance and design effective interventions to reverse the emotional distress caused by COVID-19.

## 1. Introduction

The World Health Organization (WHO) declared COVID-19 to be a global pandemic on 11 March 2020. A year and a half later, at the end of October 2021, over 242 million cases of coronavirus have been recorded around the world, leading to 4.9 million deaths. Over 4.9 million cases and 87,082 deaths have been reported in Spain [1].

Previous studies had shown that one of the groups most affected by the COVID-19 health emergency is that of healthcare professionals (HCPs) [2,3,4,5,6]. Personal, social, organizational, and/or work-related factors play a crucial role in the exacerbation or moderation of the psychological impact that such an epidemic can have on the mental health of HCPs [6,7,8,9,10,11].

In such conditions, stress is a natural, adaptive process that permits the individual to deal with the high, continual demands they are subjected to. Nonetheless, when these demands are perceived to exceed the available resources or last over a long period of time, and when the probability of adaptive coping is insufficient, there is a greater risk of suffering distress, with its psycho-emotional consequences. The results of previous research confirm the serious nature of the emotional distress suffered by HCPs during and/or after epidemic outbreaks and highlight that many of them present other mental health symptoms, such as anxiety and depression [12,13,14,15,16,17,18,19]. Moreover, after stressful events, a stress post-traumatic growth can emerge as a psychological consequence of the pandemic [20].

Not all HCPs experience the symptoms that follow the impact of such a health emergency in the same way or to the same degree [21]. Recent studies noted that nursing professionals, along with nursing assistants, showed a higher number of psychological disorders (anxiety and/or depression) than medical practitioners [7,12,21,22,23]. Another study [24] aimed to discover whether health workers had more psychosocial problems than nonmedical practitioners during the COVID-19 outbreak. Being a woman was one of the risk factors leading to anxiety and/or depression. A recent study [25] showed that female health workers and nurses reported worse psychological health than other health service professionals. The analysis by age drew contradictory results. Some studies found the highest levels of stress among younger professionals [26,27] and older professionals [28,29] or reached nonconclusive results [30]. Among mental health workers, a number of patterns of anxiety were identified in professionals working in outpatient and inpatient services. While, in general terms, the impact of the COVID-19 emergency on anxiety levels was slight, a significant number of professionals experienced severe levels of depersonalization and anxiety [31]. Under difficult circumstances, resilience is a positive outcome as an important target of prevention and intervention [32,33]

The psychological examination and diagnosis of HCPs may provide valuable information that aids in preventing or mitigating adverse psychological reactions caused by distress [34]. 

Among the important psychological aspects to be assessed are mechanisms of cognitive emotional regulation. People who are unable to successfully regulate their emotions in situations of distress experience difficulties such as impulse control problems and/or interference in goal-focused behavior [35], which may hinder the adaptive response to the demands of the surroundings [36]; in turn, this increases the likelihood of developing some type of psychopathology [37,38,39]. Cognitive emotional regulation mechanisms are the set of processes by which individuals influence their emotions [30]. Its function is to direct the emotional response, whether positive or negative, and increase, maintain, or reduce its intensity [40,41,42]. It means being aware of the relationship among emotion, cognition, and behavior, implementing effective coping strategies, as well as the ability to self-produce positive emotions [43].

Cognitive processes play an important role among the various factors that determine emotional regulation. A range of strategies have been identified depending on how people direct their thoughts during and/or after a threatening situation. Some are linked to functional and adaptive cognitive emotional mechanisms of behavior, such as being able to obtain a perspective on situations (comparing a situation or event with similar previous experiences, thus reducing its seriousness), positive focalization (the production of pleasant, compensatory thoughts, instead of focalizing on the situation of distress), acceptance (the production of thoughts that admit acceptance of the negative event), positive reinterpretation (involving the generation of thoughts that give a positive meaning to the event that caused the distress), and/or refocusing on planning (thinking of and following a series of steps that will lead to overcoming the situation of distress). Others, however, are linked to dysfunctional and maladaptive aspects of behavior; these include rumination (thinking continually about the feelings and/or thoughts related to the situation that produced the distress), catastrophizing (exaggerating those thoughts that emphasize the negative magnitude of the event), self-blame (generating thoughts that attribute the cause of the tragic event to oneself), and/or blaming others (directing thoughts that attribute the cause of the event to others) [44]. Each cognitive emotional regulation mechanism presents differential correlates with a range of psychological aspects, among which are anxiety and depression [35]. 

Much research has found a link between the use of dysfunctional cognitive emotional regulation mechanisms (DRMs) such as rumination and the presence of mental problems [45], negative affect, and/or distress [46,47,48].

During the COVID-19 lockdown, the cognitive emotional regulation mechanisms most widely used by Spanish nurses were catastrophizing, positive focalization, and positive reinterpretation [49]. Those professionals who used functional regulation mechanisms (FRMs), such as the positive reinterpretation of the situation with the aim of changing its emotional meaning, were able to reassess the situation and look for alternative ways to cope with it adaptively [50]. However, using mainly DRMs means a greater risk of vulnerability that may lead to psychopathological symptomatology [44]. Conversely, the use of FRMs increases tolerance and/or reduces the impact of stressful events.

Making a dichotomous distinction between functional and dysfunctional cognitive emotional regulation mechanisms would be both reductionist and inaccurate [51]. Results of research note that the use of FRMs and/or DRMs also depends on the characteristics of the individual or those of the specific distress-producing event or situation. Furthermore, when faced with a stimulus or situation that causes distress, cognitive emotional regulation mechanisms can be activated individually or as a group. In this frame, FRMs can be more frequent than DRMs (or the other way around) [52,53,54].

In the context of the COVID-19 pandemic, it is necessary to protect the mental health of HCPS and to articulate effective interventions that provide psychological assistance if they show early signs of anxiety or depression to minimize the risk of developing psychiatric morbidity. This research focuses on the study of cognitive emotion regulation strategies used by medical, nursing, and nursing assistant staff, as well as porters, during the first wave of COVID-19, with the following aims:

1. To describe the functional and dysfunctional cognitive emotional regulation mechanisms used by gender.
2. To screen the main group of healthcare professionals who are candidates to receive psychological assistance based on the functional and dysfunctional cognitive emotional regulation mechanisms.
3. To determine the HCP profile of candidates for psychological assistance.

The hypothesis can be stated as follows: the HCP profile of candidates for psychological assistance based on the cognitive emotional regulation mechanisms is associated with the professional activity, psychological symptoms, and age variables, but not be related to the gender variable.

## 2. Method

### 2.1. Design

- Cross-sectional descriptive study

The demographic characteristics of the participants were as follows: the study population comprised Spanish HCPs caring for patients in hospitals, health centers, and/or socio-healthcare centers, working in places with the highest risk of contact with SARS-CoV-2. The total number of HCPs in Spain is 3.87 physicians and 5.32 nurses per 10^3^ people [55,56]. The male/female ratios are 43.81/56.19 physicians and 16.38/83.62 nurses [57,58].

Nonprobability sampling was carried out for convenience. The study was approved by the Ethics ‘Committee and Research Internal Review Board of the Universitat de Girona.

### 2.2. Procedure 

The data were collected by means of an online questionnaire from 4–10 April 2020 during the peak period of infection and fatality from coronavirus [59]. The questionnaire was disseminated via social networks (WhatsApp, Instagram, Telegram, and email) following the criteria of Di Lonardo et al. [60]. All relevant ethical principles were followed to ensure that the processing of participants’ personal data complied with their rights of access and other fundamental rights under the Spanish Data Protection Act and the Declaration of Helsinki [61,62]. Participants were invited to complete the questionnaire using the “Academic Google Forms” tool, which allows invitations to be sent online through social media. Before answering the questionnaire, participants were told how long it would take to complete (5 min), the identity of the researchers, and the aim of the study. It was also clearly stated that only doctors, nurses, nursing assistants, hospital porters and/or healthcare support staff, and health science students could answer the questionnaire. Participants were offered no incentive. All voluntarily signed an informed consent document before responding. The use of the Internet and the online survey greatly facilitated data collection during one of the first and most stressful periods of the pandemic for HCPs. However, the results derived from the convenience sample used in this study cannot be extrapolated to all Spanish HCPs.

## 3. Measures

An ad hoc questionnaire was used to collect participants’ sociodemographic data (sex, age group, and geographical location), their professional activity (nursing assistant, hospital porter, nurse, doctor, or other professionals), work unit (primary care/healthcare center, emergency room/intensive care, home/geriatric hospital, COVID-19 patient center, hospital, or other), work experience (less than 1 year, from 1 to 3 years, from 4 to 9, or 10 years or more), employment status (temporary, permanent, or other), and, if applicable, the degree to which the health crisis affected their professional health activity and/or their families.

Cognitive emotional regulation mechanisms were measured using the Cognitive Emotional Regulation Questionnaire (CERQ-18), adapted to Spanish and validated for use [63]. It consists of a Likert-type scale from 1 (almost never) to 5 (almost always). Nine cognitive emotional regulation mechanisms were assessed; these were grouped into two categories: (a) functional and adaptive mechanisms, i.e., putting in perspective; positive refocusing, acceptance, positive reinterpretation, and refocusing on plans, and (b) dysfunctional and maladaptive mechanisms, i.e., rumination, catastrophizing, self-blame, and blaming others. Results of the CERQ-18 were interpreted as follows: functional and adaptive mechanisms, range from 5–25; dysfunctional and maladaptive mechanisms, range from 4–20. A higher score in the functional emotional regulation category denotes a greater functional or adaptive response. A higher score in the dysfunctional emotional regulation mechanisms implies a more dysfunctional and maladaptive type of response. Each mechanism of the questionnaire CERQ-18 includes two questions. Every question has a score ranging from 1–5. In this way, the result of the mechanism has a score ranging from 2–10. The CERQ-18 has shown sound internal consistencies across all subscales, ranging from 0.78 to 0.90 [44,64,65].

The internal consistency of the questionnaire in the sample of the study for CERQ-18 was a Cronbach’s alpha coefficient = 0.81.

In addition, the study also identified symptoms of anxiety and depression using the Spanish-validated versions of the Generalized Anxiety Disorder scale (GAD-7) (range, 0–21) [66], and the nine-item Patient Health Questionnaire (PHQ-9; range, 0–27), respectively [67]. The two questionnaires were adapted to Spanish and showed very promising metric properties, such as acceptable internal reliability. 

The cutoff points were applied as follows: GAD-7, to diagnose anxiety, was scored as 0–9, absence of anxiety, and ≥10 points, a positive screening following an in-depth interview with a clinical psychiatrist. In the case of PHQ-9, the range for diagnosing depression was scored as 0–4, absence of depression, and ≥5, the presence of depression. These measurements were based on values established by the scientific literature [4,68,69]. A description of the symptoms of anxiety and depression was published in a previous manuscript [7].

### 3.1. Main Group Candidates to Receive Psychological Support (Screening)

Firstly, DRMs were used in proportion with FRMs. Then, in order to establish the criteria of prioritization of the participant candidates for assistance, the difference between functional and dysfunctional regulation mechanisms was applied, such that a result ≤0 corresponded to the subgroup of candidates suitable for monitoring and, if need be, psychological assistance, whereas a result >0 corresponded to the subgroup where support was not prioritized.

### 3.2. Data Analysis

Data from 1481 participants were collected. Data quality management was ensured through a data-cleaning process prior to analysis. A guided data repair framework was applied: screening, diagnosing, and repairing inconsistencies. The aim was to guarantee that the data were accurate, consistent, and clear before conducting the analysis; unclean data can ultimately distort the results. The process involved running a preliminary analysis and crosschecking unexpected results against the data in the questionnaires. Any invalid, suspect data or apparently nonsensical values and duplicated rows of data were eliminated. Moreover, questionnaires with over 2% of the items unanswered (missing data) were considered invalid and consequently eliminated from the dataset in order to resolve this inconsistency and ensure greater analytical quality. This data-cleaning approach with the blank data provided both quality control and external quality. At the end of this process, 29 (2%) participants were discarded, and data from 1452 participants were considered valid for analysis.

A range of statistical inference techniques were used. Percentages and absolute frequencies were used to describe the sociodemographic characteristics and physical symptoms of the sample. Means with standard deviations (SD) were used to describe the functional and dysfunctional cognitive emotional regulation mechanisms. A Student’s *t*-test (*t* student) was used to determine the differences between the means of functional and dysfunctional mechanisms by gender. A one-way ANOVA was conducted to verify differences by professional activity. Chi-square tests were applied to identify the relationship between the categorical variables (sex, age groups, coexistence, professional experience, professional activity, anxiety, and depression) and candidates for psychological support. Logistic regression models were used to analyze associations of sociodemographic and behavioral variables with candidates to receive psychological support. The covariates included in the multivariate analysis were those that were found to be statistically significant in the univariate analysis. The Hosmer–Lemeshow test was used for analyzing goodness of fit. Considering the present sample size, a normal distribution of the variables was assumed. Statistical analyses were performed using Stata software (version 11.1, College Station, TX, USA).

## 4. Results

The final sample of HCPs who participated in the present study was *N* = 1452: 82.9% (1204) were female, and 17.1% (248) were male; 32% (465) of participants were over 50 years old, 30.5% (442) were between 40 and 49 years old, 23.5% (341) were between 31 and 39 years old, and 14% (204) were under 30; 87% (1263) of the participants originated from Catalonia, while the remainder came from other autonomous regions in Spain; 44.7% were nursing staff (649), 26.4% (383) were medical professionals, 11.5% (167) were nursing assistants, 1% (14) were hospital porters, and the remaining 16.4% (204) had other professions (pharmacists, physical therapists, residents, and students of health sciences). A total of 73.3% (1064) reported having over 10 years of professional experience, and 73.1% (1061) gave their employment status as permanent. Lastly, 43.6% (633) worked between 7 and 8 h per shift, 24.9% (362) worked between 10 and 12 h, 16.7% (242) worked more than 12 h, and 14.8% (215) worked between 8 and 10 h per shift.

### 4.1. Description of Cognitive Emotional Regulation Mechanisms Used by Healthcare Professionals (HCPs) in Relation to the Gender Variable

Considering the total sample (*N* = 1452), the results showed that acceptance received the highest score in FRMs, while rumination was higher in DRMs. Moreover, significant differences were found by the gender variable, with females scoring significantly higher than males in acceptance (FRMs), rumination, and catastrophizing (DRMs) (see Table 1).

### 4.2. Screening of the Main Group of HCPs Who Are Candidates to Receive Psychological Support

The chi-squared test was used to prioritize those candidates most suitable for therapy according to their professional activity. Considering the difference between functional and dysfunctional cognitive emotional regulation mechanisms, the results showed that 1343 participants (92.5%) were not candidates for psychological assistance, while psychological support would be prioritized in the case of 109 (7.5%). The screened group comprised 59 nurses (54.1%), 18 nursing assistants (16.5%), 17 other professionals (15.6%), and 14 doctors (12.8%) (*p* = 0.011).

A multiple-comparison test revealed that nurses and nursing assistants presented lower scores than medical professionals (Bonferroni test = −0.77, *p* ≤ 0.009; Bonferroni test = −0.1.28, *p* ≤ 0.001). Moreover, nursing assistants presented lower scores than other HCPs (Bonferroni test = −0.1.15, *p* = *0*.017).

### 4.3. Candidates for Psychological Support According to Sociodemographic, Professional, and Psychological Variables

The chi-squared test was used to identify differences between the categorical covariates in both groups (no psychological support versus psychological support). The results showed that age group, professional activity and the psychological symptoms of anxiety and depression were statistically significant between both groups (see Table 2). However, gender, years, of professional experience and living alone or with someone else were not statistically significant. 

**Figure 1:**
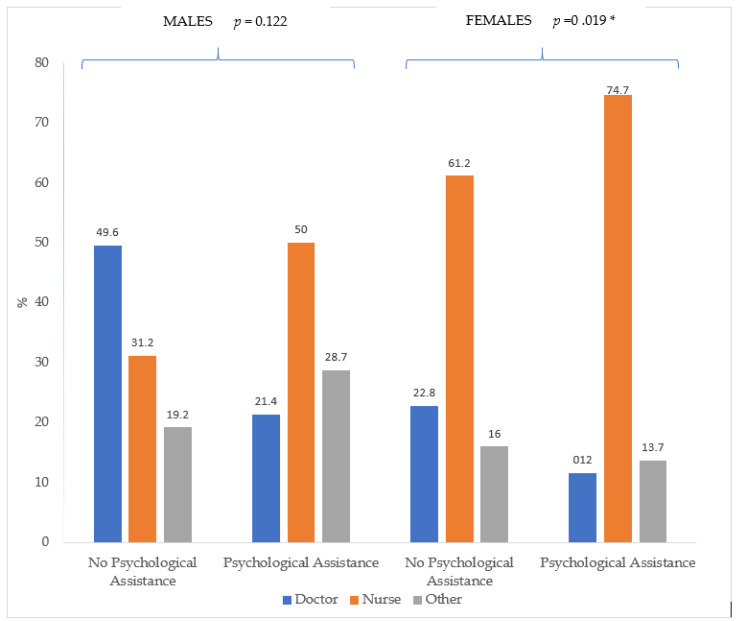
Candidates for psychological assistance according to professional activity and gender variables. * *p* > 0.001.

Figure 1 shows statistically significant differences in the subgroup of females in relation to professional activity and psychological assistance variables. Nurses accounted for 61.2% of the females who were not candidates for psychological assistance, while accounting for 74.7% of females who were candidates for assistance. On the other hand, 22.8% of those females who were not candidates for psychological assistance were doctors, as well as 11.6% who were candidates. The male subgroup showed a similar tendency to that of the females, but the absolute percentages were lower and the comparation between the three categories of professional activities was not statistically significant.

The multivariate logistic regression analysis revealed those variables that independently increase the probability of requiring psychological support. The statistically significant variables were being 30–39 years old (OR = 2.69; 95% CI: 1.26–5.75), being a nurse (OR = 2.28; 95% CI: 1.20–4.33), having anxiety (OR = 3.76; 95% CI: 2.08–6.78), and having depression (OR = 2.99; 95% CI: 1.48–6.06). Furthermore, the gender variable was not found to be an independent factor related to psychological assistance (see Table 3 and Table 4).

## 5. Discussion

There is evidence that, since the outbreak of the COVID-19 pandemic, healthcare professionals have had a high risk of suffering psychological consequences, such as symptoms of distress, anxiety, and depression [3,5,6,7,8,9,10,11,70,71]. The epidemiological data confirm that the past year has seen a rise in the prevalence and severity of these psychological symptoms among this group [20,33,72].

This study shows the result of a mental health screening strategy based on cognitive emotional regulation strategies, the aim being to detect groups of health professionals at psychological risk. The screening process took into account two interrelated and complementary aspects: (a) describing the incidence of the number of cases and their percentages, and (b) studying the severity of the mental symptoms.

The results show that the HCP profile of candidates for psychological support comprises being a nurse or nursing assistant, aged between 30 and 39 years old, and suffering from psychological symptoms of anxiety or depression. However, sociodemographic aspects such as gender, years of professional experience, and living alone or with someone else were not found to be relevant to receiving psychological assistance. That being said, other studies have confirmed that being a female is a determining variable in the profile of the candidate [70,73].

Of the 1452 health professionals who took part in the study, the (mental health) screening based on cognitive emotional regulation mechanisms confirmed that 109 (7.5%) HCPs were candidates for psychological support and should be prioritized. These results are in line with those of Lamiani et al. [74], who found that 17% of HCPs were candidates for psychological support after mental screening. Detecting those people at risk of suffering psychological disorders allows for early and effective tailored psychological intervention [75]. Furthermore, it also reduces the long-term risk of HCPs suffering other mental health disorders [76]. 

In accordance with the results of other investigations [5,70] and taking the professional activity into consideration, this study confirms that the incidence and percentage of nurses and nursing assistants who are candidates for psychological assistance is far higher than that of the other professional groups studied. The results show that the most affected group was that of nurses, with 59 (54.1%) cases, followed by nursing assistants with 18 (16.5%), and other professionals with 17 (15.6%), while just 14 (12.8%) of doctors were candidates for psychological support.

In addition, considering the severity/intensity of psychological symptoms when studying the difference between functional and dysfunctional cognitive emotional regulation mechanisms, the results confirm that nursing assistants present the lowest average value, followed by nurses. This means that these two groups have less ability to cope with distress than doctors, who show a higher average value and, thus, a greater adaptive response ability and lower risk of suffering from any associated mental disorder. It should be taken into account that nurses and nursing assistants have been permanently on the frontline, and this prolonged contact with patients may have led to greater distress and a reduced adaptive ability [6,7,8,9,10,11,20,21,22,23,33]. In a situation as drawn out as this, HCPs and mainly nurses and nursing assistants are particularly susceptible to mental health consequences such as anxiety and depression [12,13,14,15,77,78].

Furthermore, it should be emphasized that the results of this study confirm a positive relationship between DRMs and anxiety and depression [15,49,79]. A total of 77.6% of the main group of candidates for psychological support were observed to show signs of anxiety, while 87% showed signs of depression. These percentages are higher than those identified in a prior study, which detected signs of anxiety in 44.6% of participants and depression in 50.4% [70]. That being said, the percentages of anxiety and depression observed by Shahin et al. [80] were similar to those of this study, at 60.2% and 77.6%, respectively. These results corroborate the idea that the mental health symptoms suffered by HCPs during the pandemic may be both under-reported and under-treated [81].

Despite the fact that the gender variable was not found to be relevant in the description of the profile of candidates for psychological support, significant differences were observed in the female subgroup of candidates for psychological assistance according to professional activity. The percentage of female nurses who were candidates for psychological assistance was higher than that of female nurses who were not candidates for said assistance, while the percentage of female doctors who were candidates for psychological assistance was lower than the percentage of those who were not candidates to receive psychological support.

As for the male subgroup, the identified incidence was the same as that of the female subgroup, despite the fact that the absolute percentage was lower and the difference within the three male professional activities not statistically significant. These findings confirm that professional activity is a determining factor with regard to the profile of HCPs who require psychological assistance.

Assessing the coping strategies used by female HCPs to manage distress is an important element in the comprehensive assessment of mental pathologies suffered during this period. It is noted that the most common FRM used by female is acceptance and the most common DRMs are rumination, followed by catastrophizing and blaming others. These results are in line with those obtained by Giménez et al. [49]. 

According to Bonanno and Burton [82], our results showed that FRMs and DRMs are a continuance of emotional self-regulation, since an individual health professional may use a range of strategies, whether functional or dysfunctional. According to Kobylińska and Kusev [54], the effectiveness of specific emotional regulation strategies depends on contextual factors and the will of an individual to manage them in the specific context they occur.

Regarding age group, of the candidates to receive psychological support, 40 cases (36.7%) were aged between 30 and 39 years old. Our results do not coincide with those of previous studies, which suggest that younger [26,27] or older professionals are the most affected by stressful situations [28,29]. This could be explained by the stage of the life cycle in which this group finds itself, generally with young children and elderly parents and, therefore, a greater vulnerability to the personal impact of COVID-19.

A recent bibliographic review and meta-analysis [73] confirmed the results of our study that being a nurse and using maladaptive coping strategies are some of the risk factors associated with psychological distress among HCPs. Conversely, resilience contributes to adaptive coping and decreases the negative emotional reactions experienced [32,33,83].

The evaluation of psychological coping profiles can allow for the early identification of those HCPs who are most vulnerable to the presence of possible psychopathologies, as well as facilitating proactive therapeutic interventions compensating the DRMs by empowering the functional ones [84]. It also allows for optimal management of the financial and human resources needed to reduce the risk of the psychological consequences suffered by HCPs [85,86].

This study had some limitations. The study implemented a cross-sectional design; hence, causality between the variables studied cannot be confirmed. The use of convenience sampling based on social media, with targeted email communication, is susceptible to a methodological limitation in estimated response rate, response bias, and external validity. A higher percentage of nursing staff (44.7%) was represented in the sample, compared to medical professionals (26.4%) or nursing assistants (11.5%). Moreover, 82.9% of the sample comprised female HCPs, which is broadly in line with the demographics observed in hospitals based in our territory [57,58]. However, the overrepresentation of females could have affected the statistical power of the tests applied. Repeated measurements will be needed to identify any potential long-term effects of the COVID-19 pandemic.

The results of this investigation in order to screen the main group of HCPs who are candidates to receive psychological assistance could not be directly related to COVID-19 pandemics, and other factors could be associated with the main group screened. Further research is needed in Spain and internationally in order to contrast the epidemiological findings.

Lastly, the mental health screening based on cognitive emotional regulation mechanisms has proven an effective strategy for detecting the main group of HCP candidates for psychological assistance during the pandemic period. However, more work is needed to identify the factors associated with the use of dysfunctional over functional cognitive emotional regulation mechanisms and to confirm their relationship with negative mental health outcomes.

## 6. Conclusions

The main contribution of this study was a determination of the profile of those health professionals who display a greater vulnerability to psychological disorders. These results are useful in detecting those at greater risk, so that they may then receive early psychological assistance to prevent future mental health problems.

The profile of HCPs who are candidates to receive psychological support is as follows: being a nurse or nursing assistant, aged between 30–39 years old, and suffering symptoms of anxiety or depression. According to the results of this study, the gender variable is not a determining variable for this profile.

The mental screening approach has proven useful in discovering those HCPs who are most at risk and how they have dealt with this risk during the pandemic period. These results provide strong evidence for tailoring a psychological intervention adapted to HCPs who are greatly affected and suffering from more severe psychological symptoms. Therefore, monitoring and providing appropriate support should continue beyond the outbreak period to ensure a complete mental health recovery.

Dysfunctional cognitive emotional regulation mechanisms are modifiable factors, and offering HCPs monitoring and psychological support will, therefore, foster FRMs and improve the emotional distress from which they suffer.

The results of this study indicate the importance and urgency of managing mental health symptoms such as anxiety and depression observed in HCPs and offering effective psychological support to reverse the situation. 

The final suggestion of this research is the need for an institutional policy that fosters mental health among HCPs and prevents mental disorders. This requires the sustained implementation of Mental Health screening of HCPs in order to initiate an early psychological intervention. This will allow optimal management of the financial and human resources needed to reduce the lasting psychological consequences suffered by this group of HCPs on the COVID-19 pandemic continuum.

Policy plans are needed to address the mental health consequences of the pandemic. These plans should be cross-sectoral, combining mental health protection and promotion measures with actions for the treatment of additional mental illnesses.

HCPs, as well as other frontline professionals who have experienced very high levels of psychological distress during the crisis, could benefit from measures such as the use of online psychological first aid training, brief group psychological interventions, priority and specific mental healthcare for individuals/groups identified as being at risk by the screening process, and the extension of face-to-face psychological interventions with online interventions.

These actions imply a greater and better investment in mental health services for the benefit of the HCP community. Mental health and psychosocial support needs to be included in coordination and planning programs, both now and in the long term. Mental health will remain a key concern even as countries emerge from the pandemic and embark on social and economic recovery.

## Figures and Tables

**Table 1:** Functional and dysfunctional cognitive emotional regulation mechanisms used by HCPs.

Cognitive Emotional Regulation Mechanisms	Total	Male	Female	*p*-Value
	*N* = 1452	*N* = 248	*N* = 1204	
	Mean ± SD	Mean ± SD	Mean ± SD	
Functional				
Putting into perspective	5.44 ± 1.68	5.47 ± 1.63	5.43 ± 1.69	0.28
Positive refocusing	5.67 ± 2.04	5.76 ± 1.98	5.65 ± 2.05	0.755
Acceptance	7.52 ± 2.08	7.18 ± 2.28	7.58 ± 2.03	0.006 *
Positive reappraisal	7.33 ± 2.08	7.35 ± 2.03	7.33 ± 2.09	0.444
Refocus on planning	6.88 ± 1.91	6.67 ± 1.91	6.92 ± 1.9	0.44
Dysfunctional				
Rumination	5.5 ± 1.93	5.2 ± 1.97	5.56 ± 1.92	0.008 *
Catastrophizing	5.27 ± 2.20	4.61 ± 2.09	5.41 ± 2.20	≤0.001 *
Self-blame	2.52 ± 0.98	2.56 ± 1.03	2.52 ± 0.98	0.59
Other-blame	4.58 ± 2.48	4.59 ± 2.64	4.58 ± 2.45	0.094

* Statistically significant according to independent sample *t*-test (Student). Similar variations are assumed. Levene’s test (*p* > 0.05).

**Table 2:** Sociodemographic and professional characteristics of HCPs.

Variables	Total
	*N* = 1452
	*n* (%)
Gender	
Male	248 (17.1)
Female	1204 (82.9)
Age (years)	
<30	204 (14.0)
30–39	341 (23.5)
40–49	442 (30.4)
≥50	465 (32.0)
Geographical location	
Catalonia	1263 (87)
Rest of Spain	189 (13)
Coexistence	
Alone	166 (11.4)
Someone else	1286 (88.6)
Professional experience (years)	
<1	33 (2.3)
1–3	127 (8.7)
4–9	228 (15.7)
≥10	1064 (73.3)
Professional activity	
Nursing assistant	167 (11.5)
Nurse	649 (44.7)
Doctor	383 (26.4)
Hospital porter	14 (1)
Other	239 (16.5)
Work unit	
Primary care/health care	216 (14.9)
Intensive care/emergency room/	287 (15.9)
Geriatric hospital/socio-sanitary residence	70 (4.8)
COVID-19 patient center	110 (7.6)
Hospitalitzation	702 (55.8)
Other health centers	67 (4.6)
Employment status	
Temporary contract	289 (19.9)
Permanent contract	1061 (73.1)
Other	292 (7)
Hours of work per shift	633 (43.6)
Between 7 and 8 h	215 (14.8)
Between 8 and 10 h	362 (24.9)
Between 10 and 12 h	242 (16.7)
>12 h	
Psycghological symptoms	
Depression (*n* = 1308)	
Absence of depression	624 (43)
Presence of depression	684 (47.1)
Anxiety (*n* = 1402)	
Absence of anxiety	844 (58.1)
Presence of anxiety	558 (38.4)

**Table 3:** Candidates for psychological assistance according to sociodemographic, professional and psychological variables.

Variables	Total	No Psychological Assistance	Psychological Assistance	*p*-Value
	*N* = 1452	*N* = 1393	*N* = 109	
	*n* (%)	*n* (%)	*n* (%)	
Gender				
Male	248 (17.1)	234 (17.4)	14 (12.8)	0.222
Female	1204 (82.9)	1109 (82.6)	95 (87.2)	
Age (years)				
<30	204 (14.0)	191 (14.2)	13 (11.9)	
30–39	341 (23.5)	301 (22.4)	40 (36.7)	0.009 *
40–49	442 (30.4)	413 (30.7)	29 (26.6)	
≥50	465 (32.0)	438 (32.6)	27 (24.8)	
Coexistence				
Alone	166 (11.4)	152 (11.3)	14 (12.8)	0.630
Someone else	1286 (88.6)	1191 (88.7)	95 (87.2)	
Professional experience (years)				
<1	33 (2.3)	32 (2.4)	1 (0.9)	
1–3	127 (8.7)	118 (8.8)	9 (8.3)	0.457
4–9	228 (15.7)	206 (15.3)	22 (20.2)	
≥10	1064 (73.3)	987 (73.5)	77 (70.6)	
Professional activity				
Nurse *	830 (57.1)	752 (56.0)	78 (71.6)	0.002 *
Doctor	383 (26.4)	369 (27.5)	14 (12.8)	
Other	239 (16.5)	222 (16.5)	17 (15.6)	
Anxiety (*n* = 1402)				
No	844 (60.2)	820 (63.3)	24 (2.4)	≤0.001 *
Yes	558 (39.8)	475 (36.7)	83 (77.6)	
Depression (*n* = 1308)				
No	624 (47.7)	612 (50.3)	12 (13.0)	≤0.001 *
Yes	684 (52.3)	604 (49.7)	80 (87.0)	

* Including nursing assistants and hospital porters.

**Table 4:** Candidates to receive psychological support. Association with sociodemographic, professional, and psychological variables.

Variables	Univariate OR (95% CI); *p*-Value	Multivariate OR (95% CI); *p*-Value
Gender		
Male	1	1
Female	1.43 (0.80–2.55); 0.224	0.80 (0.41–1.55); 0.503
Age (years)		
<30	1	1
30–39	1.95 (1.02–3.74); 0.044	2.69 (1.26–5.75); 0.011 *
40–49	1.03 (0.52–2.02); 0.928	1.76 (0.81–3.81); 0.154
≥50	0.90 (0.46–1.79); 0.776	2.17 (0.98–4.80); 0.057
Professional activity		
Doctor	1	1
Nurse	2.73 (1.53–4.89); 0.001	2.28 (1.20–4.33); 0.012 *
Other	2.02 (0.97–4.17); 0.058	1.77 (0.77–4.08); 0.180
Anxiety (*n* = 1402)		
No	1	1
Yes	5.97 (3.74–9.53); ≤0.001	3.76 (2.08–6.78); ≤0.001 *
Depression (*n* = 1308)		
No	1	1
Yes	6.75 (3.64–12.5); ≤0.001	2.99 (1.48–6.06); 0.002 *

* Including nursing assistants and hospital porters.

## Data Availability

Not applicable.
